# Mobile Therapy: Case Study Evaluations of a Cell Phone Application for Emotional Self-Awareness

**DOI:** 10.2196/jmir.1371

**Published:** 2010-04-30

**Authors:** Margaret E Morris, Qusai Kathawala, Todd K Leen, Ethan E Gorenstein, Farzin Guilak, Michael Labhard, William Deleeuw

**Affiliations:** ^3^Behavioral Medicine ProgramDepartment of PsychiatryColumbia UniversityNew York, NYUSA; ^2^Division of Biomedical EngineeringOregon Health and Sciences UniversityPortland, ORUSA; ^1^Digital Health GroupIntel CorporationBeaverton, ORUSA

**Keywords:** Mood phone, experience sampling method, ecological momentary assessment, cognitive behavioral therapy, affect, mood, emotion, mobile phone, self-assessment, Mood Map, cellular phone, psychotherapy, stress, technology, sampling, user centered design

## Abstract

**Background:**

Emotional awareness and self-regulation are important skills for improving mental health and reducing the risk of cardiovascular disease. Cognitive behavioral therapy can teach these skills but is not widely available.

**Objective:**

This exploratory study examined the potential of mobile phone technologies to broaden access to cognitive behavioral therapy techniques and to provide in-the-moment support.

**Methods:**

We developed a mobile phone application with touch screen scales for mood reporting and therapeutic exercises for cognitive reappraisal (ie, examination of maladaptive interpretations) and physical relaxation. The application was deployed in a one-month field study with eight individuals who had reported significant stress during an employee health assessment. Participants were prompted via their mobile phones to report their moods several times a day on a Mood Map—a translation of the circumplex model of emotion—and a series of single-dimension mood scales. Using the prototype, participants could also activate mobile therapies as needed. During weekly open-ended interviews, participants discussed their use of the device and responded to longitudinal views of their data. Analyses included a thematic review of interview narratives, assessment of mood changes over the course of the study and the diurnal cycle, and interrogation of this mobile data based on stressful incidents reported in interviews.

**Results:**

Five case studies illustrate participants' use of the mobile phone application to increase self-awareness and to cope with stress. One example is a participant who had been coping with longstanding marital conflict. After reflecting on his mood data, particularly a drop in energy each evening, the participant began practicing relaxation therapies on the phone before entering his house, applying cognitive reappraisal techniques to cope with stressful family interactions, and talking more openly with his wife. His mean anger, anxiety and sadness ratings all were lower in the second half of the field study than in the first (*P* ≤ .01 for all three scales). Similar changes were observed among other participants as they used the application to negotiate bureaucratic frustrations, work tensions and personal relationships. Participants appeared to understand the mood scales developed for this experience sampling application and responded to them in a way that was generally consistent with self-reflection in weekly interviews. Interview accounts of mood changes, associated with diurnal cycles, personal improvement over the course of the study, and stressful episodes, could be seen in the experience sampling data. Discrepancies between interview and experience-sampling data highlighted the ways that individuals responded to the two forms of inquiry and how they calibrated mood ratings over the course of the study.

**Conclusions:**

Participants quickly grasped the Mood Mapping and therapeutic concepts, and applied them creatively in order to help themselves and empathize with others. Applications developed for mobile phones hold promise for delivering state-of-the-art psychotherapies in a nonstigmatizing fashion to many people who otherwise would not have access to therapy.

## Introduction

Emotional self-awareness is an important skill for personal health [1,2]. Awareness of emotional patterns helps people recognize the situational nature of distress, and over time, to modulate their reactions to stressful events. The ability to monitor and modulate emotional reactions, that is, self-regulation, impacts both mental and physical health [3]. Particularly important for cardiovascular health is emotional resilience, that is, the ability to bounce back quickly from a stressful event [4]. Emotional awareness and self-regulation are becoming recognized as societal issues due to the influence of mood on physical health, interpersonal relationships, civic engagement, and professional effectiveness [5]. Public policy campaigns have called for extending psychotherapy to the large numbers of people who typically do not have access to such services [6,7].

Personal technologies hold promise for helping people learn about their emotional patterns and improve coping in the flow of daily life. Here, we explore mobile phones for extending the well-evidenced practice of cognitive behavior therapy, in which patients learn to modify thought and behavior patterns that contribute to negative emotional states [8]. The appeal of mobile phones as a vehicle for therapy lies in their relatively low cost and lack of stigma, as well as their ability to capture data and offer coaching throughout the day. Experience sampling, a method for prompting in-situ self-report, is well suited for deployment on mobile phones [9]. This technique has been applied previously to understand how a wide range of behaviors and moods vary over time, place, and situations [10]. Prompting at random intervals addresses reporting biases, such as the tendency to recall the most intense and recent emotions [9,11]. Historically, beepers and journals have been the primary tools for experience sampling, but these are cumbersome for long-term use. More recently, Twitter and other microblogging tools accessible through mobile devices, have been allowing many people to sample and share their own behaviors, feelings, and experiences throughout the day.

**Figure 1:**
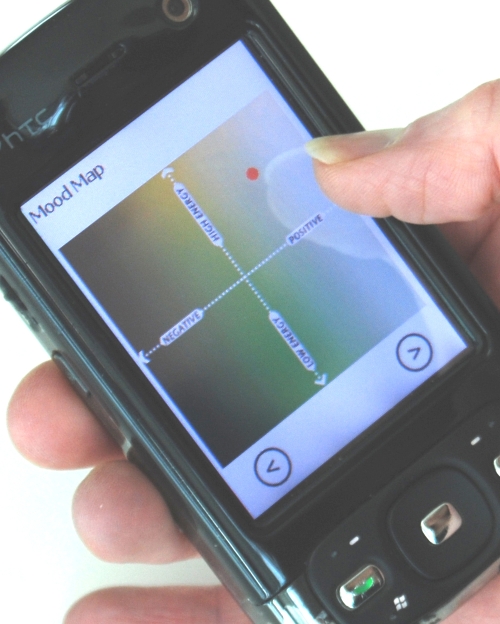
Mood Map

**Figure 2:**
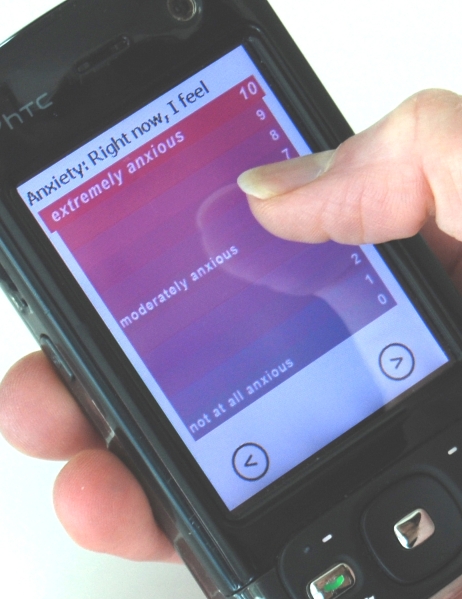
Single-dimension mood scale

In this study, we examined the use of a mobile phone application that combined experience sampling of mood with exercises inspired by cognitive behavioral therapy. The intent of the technology was twofold: to gather data trends that could be illuminating to individuals over time and to offer some interventions that could be activated on the spot. This study emerged from a project called Mobile Heart Health, in which mobile therapies were triggered by physiological indications of stress [12]. The physiological sensors, which detected stress according to changes in heart rate variability, were compelling but not easily deployable for extended field trials. Two parts of the Mobile Heart Health system—the experience sampling and the therapies—were tested in the field studies described in this paper. We designed a touch screen Mood Map and several single-dimensional mood scales (Figure 1 and Figure 2) to invite self-reflection and minimize the burden of self-reporting. The Mood Map allowed participants to plot their moods on a two dimensional space, according to their level of physical energy and the valence of their emotional state. This interface was based on the circumplex model, which describes all emotion by these two factors and has been shown to account for 70% of the variance in self-reported affective states [13].

The mobile therapies delivered in our current study were inspired by cognitive therapy interventions. They included visualizations for physical relaxation and cues for cognitive reappraisal. In cognitive reappraisal, patients monitor and correct “attributional biases” in their automatic reactions or interpretations [14]. Biases that have been associated with negative affect include attributing negative events to internal, permanent and global causes, and exaggerating the urgency and morality of situations [15,16]. The reappraisal techniques used to challenge these biases inspired the Mind Scan exercises, one of the mobile therapies described below.

## Methods

### Participants

Of the ten adults who enrolled in the study, 6 were women and 4 were men. Participants’ ages ranged from 30 to 48 with a mean age of 37 years (SD 5.75). Of these participants, eight completed the study and two discontinued participation due to time constraints. Participants were employees at a large corporation. All had completed a Mayo Clinic Health Risk Assessment in which they indicated a stress level of 3 or above on a 5-point scale. Health coaches showed notices about the study to people they thought might be interested in participating. The notices instructed those who were interested to contact the researcher for more information about the study.

### Materials

The mood sampling application was run on HTC 3600 mobile phones provided to participants for use during the study. The phones could not be used for phone calls during the study because of complications associated with transferring calling plans. In addition to a phone, each participant was given a charger and a manual that provided detailed instructions and contact information for technical support.

The application consisted of mood reporting scales and mobile therapies. The mood reporting scales included the Mood Map and single-dimension mood scales for happiness, sadness, anxiety, and anger. All scale entries were made via the touch screen. The application logged the time and date of all user interactions.

The Mood Map is a touch-screen translation of the circumplex model of emotion [13], shown in Figure 1. Participants described their mood by indicating its location on a two-dimensional space formed by the horizontal axis of “negative–positive” and the vertical axis of “high–low” energy. The left endpoint of the x-axis, valence, is labeled “negative,” and the right endpoint is labeled “positive.” The bottom end of the y-axis, arousal, is labeled “low energy,” and the top end, “high energy.” When an individual places a fingertip on the appropriate location on the Mood Map, a red dot appears to indicate his or her mood at the time of the experience sampling inquiry. Each axis was intended to capture 15 discrete values, from -7 to +7. An error in data capture limited analysis of the x-axis to a bipolar distinction (ie, whether moods were positive or negative), but the full spectrum of y-axis values, that is, energy ratings, were captured accurately.

Single-dimension mood scales for anger, anxiety, happiness, and sadness were used in addition to the Mood Map. These measures of specific emotions complemented the general expression of emotional experience captured by the Mood Map. These mood scales, adapted from a rating scale by Brown [17], were arranged vertically on the screen, with an 11-point range. They were labeled with the specific emotion and were graded in color between the two endpoints. An example, the anxiety scale, is shown in Figure 2. Each scale proceeded from “not at all” (followed by the mood being captured, such as “anxious”) at the bottom of the screen, to “extremely” (again followed by the particular mood) at the top of the screen. The experience sampling application pushed these scales to participants at scheduled times throughout the day.

Participants were prompted for their moods in the morning, evening and throughout the day, using an experience sampling program called MyExperience [18]. The times of morning and evening prompts were determined in the introductory interview, based on each participant’s daily habits and schedules. Participants could select prompting intervals ranging from 30 minutes to three hours, but the exact time at which the prompt appeared within this interval varied by several minutes to prevent prepared responses.

To reduce monotony and mindless responses, the prompts alternated between a long and short set of questions. The long query consisted of the Mood Map and all four mood scales: anxiety, anger, happiness and sadness. The short query consisted of the Mood Map and one single dimension mood scale. The single dimension mood scale in the short version depended on the quadrant of the Mood Map response. For example, a Mood Map response in the upper left quadrant (negative mood, high energy) was followed by the anxiety scale, whereas a Mood Map response in the upper right quadrant (positive mood, high energy) was followed by the happiness scale. Participants were instructed to ignore prompts that could disrupt their work or personal communication (eg, a mood query during an important meeting).

Individuals varied considerably in the frequency of their responses: Over the course of the study, the number of mood scale responses ranged from 412 to 828 with a median of 612. Most participants used the application in spurts rather than steadily. On average, participants completed 21 mood scales per day.

**Figure 3:**
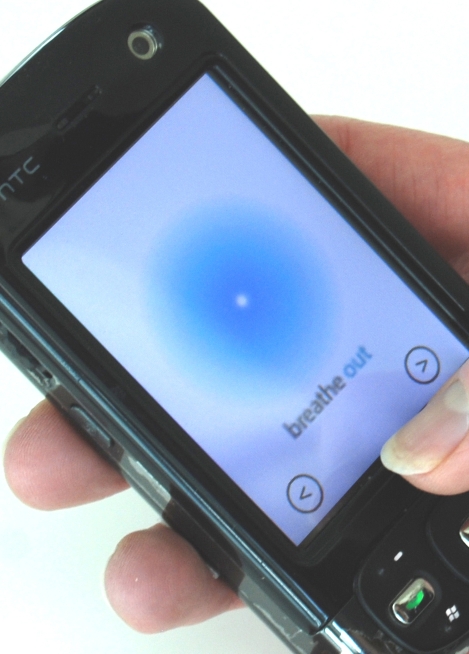
Breathing exercise

**Figure 4:**
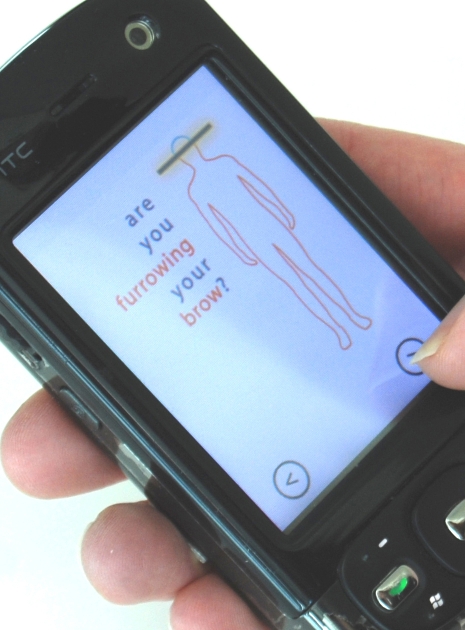
Body Scan

**Figure 5:**
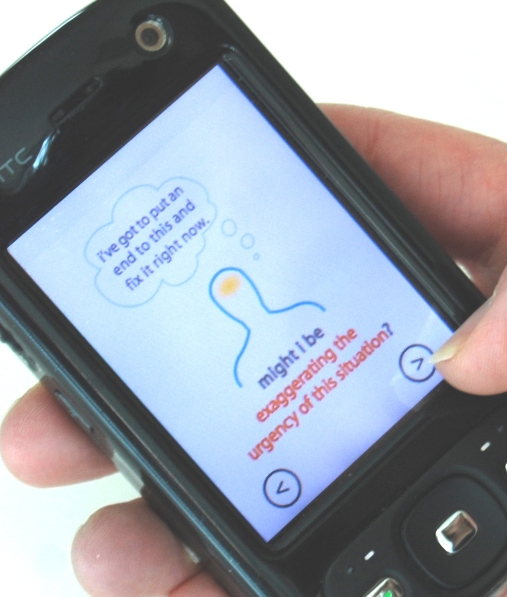
Mind Scan

Once participants recognized their moods, they could access the “mobile therapies,” short translations of cognitive behavioral therapy concepts adapted to the mobile phone (shown in Figure 3, 4, and 5). These could be activated by touching icons on the main screen of the application. The principal mobile therapies included a breathing visualization (Figure 3), a physical relaxation animation called the Body Scan (Figure 4), and a series of cognitive reappraisal exercises called the Mind Scan (Figure 5, described below). The breathing exercise was a blue circle that expanded and contracted slowly to encourage deliberate and slower breathing. The Body Scan included an outline of a human figure with rhetorical questions about where the user might be holding tension, for example, “Are you furrowing your brow?” As the user clicked through the questions, that section of the body outline changed from red to blue.

The Mind Scan was a series of rhetorical questions designed to encourage cognitive reappraisal. The questions addressed cognitive distortions associated with depression, such as attributing negative events to global, stable and internal causes [15]. For example, one Mind Scan screen asked, “Might I be globalizing?” and was accompanied by the example thought, “It’s not just that report; it’s my whole career.” Other distortions, identified by Gorenstein and colleagues [16], related to the perception of annoyances as injustices, translated as, “Might I be making rules out of my pet peeves?” and an exaggerated sense of urgency to resolve an issue, that is, “Might I be exaggerating the urgency of this situation?” Participants could activate the breathing, Body Scan, and Mind Scan features directly or could select “coaching” to access a series of visual prompts for effective handling of interpersonal conflict. All of these exercises could be completed in a minute or less.

### Procedures

Participants were recruited from a sample of employees who completed a Health Risk Assessment with a health coach. The health risk assessment involved an online survey from the Mayo Clinic that included a question about stress. Those who rated their stress levels as 3 or higher on a scale of 1 to 5 in response to this question were told about the study by the health coach. Potential candidates were given a flyer about the study and told that they could contact the primary researcher for more information.

In a first meeting with the researcher, the application was described and participants were screened for their availability to use the application and participate in four weekly interviews, each approximately one hour in length. Rule-out criteria included extensive travel and current involvement in psychological or psychiatric treatment. These criteria were stated in the consent form, which was read aloud to participants. Training on the phone application, including detailed guidance on Mood Mapping and mobile therapies, occurred in follow-up meetings. Participants were given a phone to use for the study, along with a detailed manual and encouragement to contact researchers with questions.

Four weekly interviews, the last of which was an exit interview, were conducted with each participant by a single researcher, a clinical psychologist. The interviews were open-ended conversations about how participants had used the phone application to reflect on their moods and handle stressful situations, and to identify other ways they had shaped the application to the nuances of their lives. Participants shared reactions to the application and to trends of their experience sampling data, which were shown on a laptop computer. The interviews were structured not as therapy sessions but as discussions about how people could interact with the application in a therapeutic way. Interviews were approximately one hour in length and were recorded and transcribed. In the initial interview, participants described sources of stress and the way stress manifested in their relationships, behaviors and physical experiences. In the exit interview, participants discussed their final week of participation and summarized their experiences with the application.

This study was approved by a board responsible for privacy considerations at the participants’ place of employment. Identifiers were removed from the data collected from participants’ phones, and data were stored in an encrypted database. Data stored on the phone (numerical responses to scales) were associated with participant numbers but not names. Analysis was conducted after the data were downloaded from participants’ phones. The entries made by participants were stored on the phone and not transmitted to anyone outside of weekly downloads. As a result—and as we explained to participants—there was no real-time monitoring of their entries, and no possibility of real-time interventions based on their reports of negative moods. Participants did not receive any financial compensation or organizational recognition for their involvement in the study. Participants were told at the outset that there was no known benefit to participating, and that they could discontinue at any time.

## Results

To understand how the phone was used for self-reflection and coping, we analyzed interview narratives and experience sampling data for indications of change in mood patterns. Accounts of mood changes from interviews were used to examine patterns in the experience sampling data during the same time ranges.

We examined changes that were described in interviews as occurring over the course of the one month study; characteristic patterns of change over the diurnal cycle; and changes during specific stressful incidents. These three categories emerged from qualitative analysis of interview data. Agreements and disagreements between experience sampling data and weekly interviews are highlighted in the case studies. A number of participants reported changes over the course of the study in their mood patterns and coping skills, and ascribed these changes to use of the application. These examples illustrate the potential of mobile tools not just for gathering data about mood patterns, but also for querying in a way that invites emotional awareness, self-regulation, and behavioral change. Five case studies are shared.

We examined changes over the one-month study in several ways. We used the Behrens-Fisher *t*-test to compare data from the first half of the study with data from the second half. We also examined standard and robust linear regression of the mood scales against time in the one-month study.The regression results largely corroborated the *t*-test results, but since the linear trends generally are not a good fit to these data, we report only the *t*-test results here.

To study diurnal patterns, we segmented each participant's data into time blocks. This segmentation was guided by the raw data and by mood patterns reported during the interviews. We used a two-way analysis of variance (ANOVA) for joint analysis of diurnal changes and changes over the course of the study. The diurnal time blocks just mentioned formed the first grouping variable for this analysis. Two groupings were explored for the second variable: (1) week number in the study, and (2) first versus second half of the study. This joint analysis of diurnal changes and changes over the course of the study allowed us to study interaction effects between the two. Bonferroni adjustments were made for multiple comparisons in the ANOVA. We adopted a significance level of *P* = .05 for reporting results and report the actual *P* values for significant test results, except when *P* < .001.

To examine stressful episodes, we segmented the participant’s experience sampling data according to the time intervals of incidents reported in interviews. We analyzed whether emotion ratings during time intervals corresponding to reported stressful episodes differed from the emotion ratings outside of those intervals, referred to as the background period. We compared the mood scale levels in the episodes with the background by one-way ANOVA. Names and identifying information of participants have been changed.

### Case Studies

#### Tobias

Tobias, a man in his early thirties, enrolled in this study because he was eager to extend his self-improvement from weight loss to stress reduction. During the previous year he had lost close to 60 pounds by following a strict diet and exercise plan. His stress stemmed from conflict with his wife over childcare and household responsibilities.

Tobias described a clear diurnal pattern in his mood. Each day at 5PM, he raced back from work to immediately take over responsibility for the kids, pets, dinner, and general chaos of home life, as his wife, also exhausted, left the house to find time alone. He found the transition jarring and often remained irritated the entire evening.

**Figure 6:**
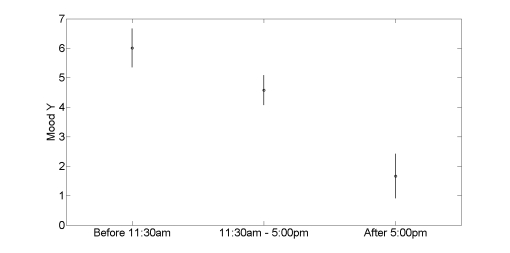
Progressive drop in Tobias’ energy through the day. The circles show the mean values in the diurnal segments indicated on the abscissa. Error bars show the 95% confidence limits on the means. Note that the total Mood Y range available to the user is [-7, +7].

Echoing this verbal account, Tobias’ phone entries show a decrease in energy upon coming home. In fact, his energy, as reflected in the Y values in the Mood Map, decreased continually throughout the day. As shown in Figure 6, his mean energy values were 6.01 before 11:30AM, dropped to 4.58 between 11:30AM and 5:00PM, and further dropped to 1.67 from 5:00PM until his last recording at 10:49PM (two-way ANOVA, *P* <  .001).

Tobias’ mood and communication patterns shifted as the study progressed. He explored some of the mobile therapy concepts, using them to anticipate his negative reactions to coming home and curtail them so that they would not dominate the entire evening. Tobias applied a rhetorical question from the Mind Scan, “Might I be stabilizing?” by telling himself “Just be prepared for the next 15 to 30 minutes…It probably isn't going to be an ideal situation for you, but just get through the 15 to 30 minutes and then, you know, you’ll be fine.” He practiced this and other short exercises before he walked in the door. Perhaps more importantly, he spoke with his wife about alternative solutions and schedules for sharing responsibilities. He was pleased by the self-awareness and coping abilities he developed during the study.

**Figure 7:**
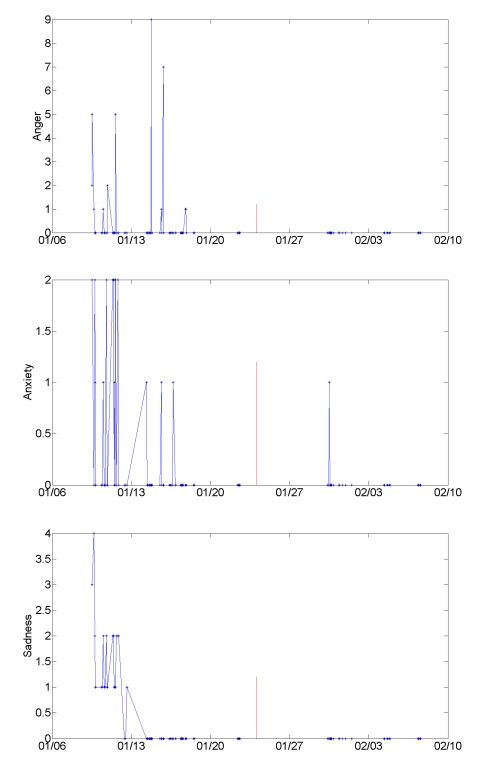
Anger, anxiety, and sadness mood ratings change across the study for Tobias, who described improved mood and better communication with his wife. Vertical lines mark the half-way point in the study.

The improvements in mood and family relations that Tobias described are reflected in his mood entries. There was a lifting of energy (the Y value of the mood scale), and a decrease in negative emotions on the single dimension scales. Figure 7 shows his anger, anxiety, and sadness ratings throughout the study. His mean anger, anxiety, and sadness ratings all were lower in the second half of the study than in the first half. His mean anger ratings decreased from 0.49 during the first half of the study to zero in the second half (*P = .*01, Behrens-Fisher *t*-test). His anxiety ratings decreased from 0.37 to 0.04 (*P* = .006), and his sadness ratings from 0.61 to zero (*P* < .001). His energy ratings (y-axis of Mood Map) increased from 3.28 in the first half of the study to 6.58 in the second half (*P* < .001).

Tobias’ pattern of decreased energy in the evenings abated to some degree as the study progressed. His energy continued to decrease throughout the day, but the decrease was less extreme in the second half of the study than in the first half. Two-way ANOVA comparing his energy before and after 4PM and between the first and second half of the study showed a significant interaction (*P =*.005). Specifically, in the first half of the study, Tobias’ energy ratings before 4PM averaged 5.09 and after 4PM averaged 2.22. In the second half of the study, his energy ratings before and after 4PM were more nearly equal (3.67 and 3.64, respectively). That is, Tobias showed less fatigue or burnout in the evening hours as the study progressed.

In addition to improved mood, Tobias described greater self-awareness throughout the study. He found the Mood Map useful as a way “to check in with myself.” At the beginning of the study, he was disappointed that the system wasn’t telling him his mood: “What I was hoping this device was going to be was something that told me how I was feeling, because that’s one of the things I struggle with,” he said. Later, though, he expressed a comfortable curiosity in his mood patterns, and “more confidence in my feelings.”

#### Theresa

Another participant whose mood improved over the course of the study was Theresa, a woman in her late thirties who had been chronically frustrated at home and in her work as a manager. Her frustration at home related to her niece, who recently had moved in with the participant but never helped with household chores and continually left the lights on when she left the house. At work, Theresa struggled with a technician on her team who failed to take ownership for finishing tasks. In an interview, she characterized her exasperation with both relationships: “It’s like [the movie] *Groundhog Day*...it’s the same thing...over and over again!”

Eventually, Theresa tried out a collaborative approach that worked well in both situations. At home, she devised a system that finally motivated her niece to turn off the lights: “I was like, ‘Okay, maybe we’ll have an energy conservation initiative with her, that if she turns off all the lights before she goes to school and turns down her heat, that’s a point. And we’ll keep points every day.’” And at work, she suggested a “priority list” for managing tasks, and this approach went over well with the technician: “He continued to work on the list, so it was working as expected...and it turns out he loves it!”

She described her satisfaction with her negotiation: “It was this neat experience for me…The conflict was done…and I didn’t grow up that way…It goes back to looking—okay, ‘What’s the priority?’ ‘What is the true goal?’ because we both have the same goal in mind, but we might get there different ways. So, I think that the questions that are on there, you know, helped to get to that, even if I didn’t look at them right before the meeting”. In these quotes, Theresa not only describes the skills that she developed during the study, but also her internalization of the concepts. She interweaves language and concepts from the mobile therapies, such as “What is the true goal?” with her self-reflection in the interview. She also makes it clear that she applies these concepts even if she isn’t looking at the phone.

**Figure 8:**
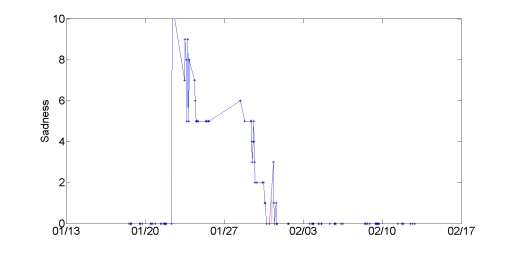
Sadness decreases dramatically in the second half of the study for Theresa, coinciding with her successful negotiation of conflict at home and work.

The satisfaction that Theresa described is reflected in the positive change in her mood ratings recorded on the phone. The energy dimension of her Mood Map ratings rose from a mean 1.14 in the first half of the study to 1.8 in the second half (Behrens-Fisher *t*-test *P* = .01). As shown in Figure 8, her sadness decreased dramatically, from a mean of 3.15 to 0.875 (*P* < .001), the largest cross-study shift in a mood scale we observed among all participants. Surprisingly, this decided drop in sadness was not accompanied by a significant drop in anger. This discrepancy suggests that the label of anger on the specific scale did not resonate with Theresa’s frustration, and points to the need to tailor mood queries to an individual’s emotional signature, that is, the range and pattern of each person’s emotions.

#### Forest

Personal stressors marked the interview and experience sampling data of Forest, a man in his mid thirties who had recently moved to the United States. He described frequent anger and frustration related to an overarching struggle to establish professional and financial stability.

Here we explore two stressful episodes described in Forest’s interviews and experience sampling data. The first stressor, which occurred early in the study, followed his wife’s selection of a physician who was not covered by his insurance. He spent days on the phone arguing with insurance companies and with the physician, trying to find a way to please his wife without incurring enormous expense. Although he and his wife eventually agreed on a doctor within the insurance network, he regretted the fruitless frustration he experienced along the way.

**Figure 9:**
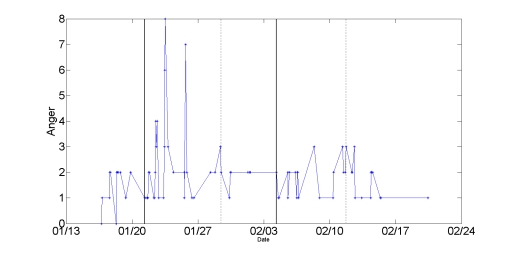
Snapshots of two comparably stressful episodes (1/21 to 1/29 and 2/4 to 2/11) identified by Forest during interviews. The solid and dotted vertical lines mark the beginning and end of the episodes respectively. In the second episode, he applied stress management and conflict resolution techniques and reported less anger.

Forest’s mean anger ratings (but not other emotion ratings) during this episode were significantly higher than the background level (2.41 vs. 1.57, *P* = .03). This difference is reflected in the time series of his anger ratings, shown in Figure 9.

Several weeks later, Forest relayed a similarly stressful series of interactions as he tried to obtain a US passport for his daughter. He was turned away because of missing paper work on his first visit to the consulate, and on each subsequent visit he had to interact with a rude officer.

**Figure 10:**
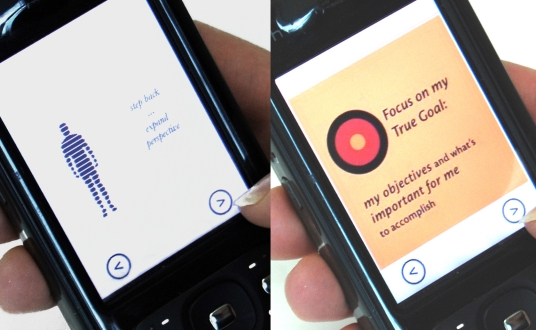
Forest and other participants quickly internalized the mobile therapies.

Although irritated, Forest mentally prepared for each follow-up interaction by repeating to himself some of the mobile therapy concepts about goal orientation and constructive confrontation. In an interview, he relayed his self-talk from the day of the incident, in which he combined text from the mobile therapies shown in Figure 10 (“Step back…expand perspective” ) with his own self-reflection ( “What is my goal here? So what if I don’t like this guy? Step back, expand perspective.”)

Even though securing the passport required a stressful series of interactions that took up far more time than he had anticipated, Forest felt good about the outcome and the way he handled the interactions. Unlike the first episode, his anger ratings associated with obtaining the passport were not significantly higher than the background level. This episode is reflected in the second segment of the anger time series (dated from 2/4 to 2/11) in Figure 9. The majority of Forest’s mobile therapy usages occurred during these two stressful episodes, suggesting that he reached out to the phone for help in moments of need.

#### Octavia

**Figure 11:**
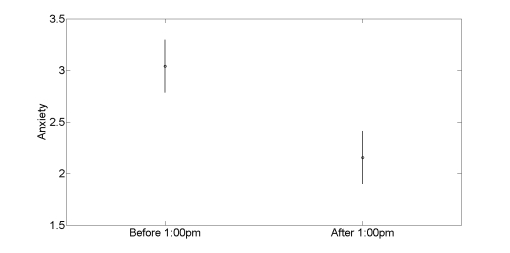
Octavia’s interview accounts of morning procrastination are paralleled in her experience sampling data, which show elevated anxiety before 1:00 PM. The circles show the mean values in the diurnal segments indicated on the abscissa. Error bars show the 95% confidence limits on the means.

Octavia, a woman in her late thirties with an advanced technical job, described ongoing struggles with anxiety and procrastination. After several reorganizations in her division, she struggled to prioritize tasks and spent much of her day simply reacting to email or addressing small requests. She described the most difficulty focusing and the most anxiety in the morning. In keeping with this interview account, her mood phone entries showed more negativity in the morning hours than in the afternoon. As shown in Figure 11, her anxiety averaged 3.04 before 1:00PM, and dropped to 2.16 after (two-way ANOVA, *P* < .001). Her unhappiness dropped from a mean of 4.27 before 1:00PM to a mean of 3.94 after (*P* = .01).

**Figure 12:**
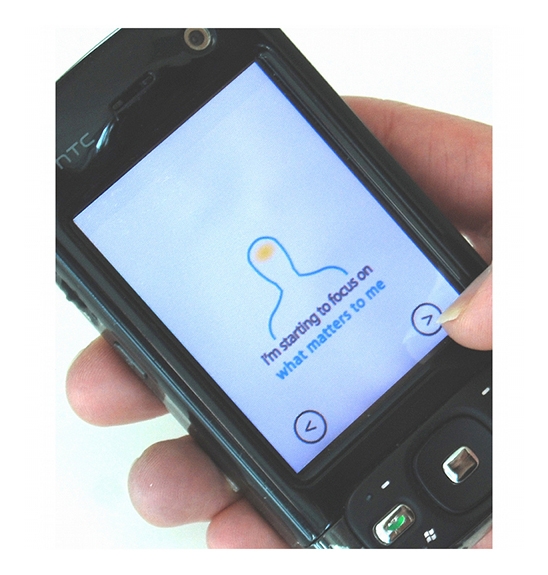
Example Mind Scan prompt that helped Octavia prioritize and stop procrastinating

In her closing interview, Octavia described notably better focus, productivity and clarity in presenting her work to others. She attributed these improvements to the phone application, particularly the prompts about prioritizing (an example prompt is shown in Figure 12):

Not a whole lot else has changed other than usage of this (application) and just a refocusing…what it helped me say is, “What is the absolute most important thing I should accomplish?”…knowing there are other things out there that need to happen, that just are not quite as important…I was thinking about the visual…where it says, “Focus on what matters to me.”

**Figure 13:**
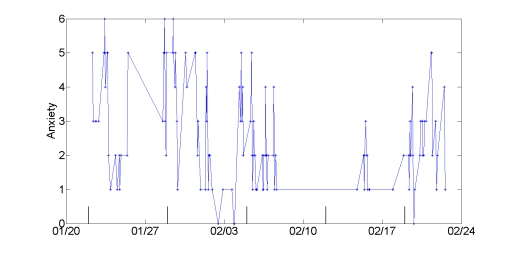
This time series visualization shows Octavia’s lowered anxiety ratings in weeks three and four of the study, a pattern that matches her interview account of decreased anxiety. The short vertical lines along the x-axis mark the beginning of each week in the study.

Octavia’s verbal account of increased focus over as the month that she used the phone application was echoed in the experience sampling data: Her mean anxiety dropped from 2.88 to 2.16 from the first to the second half of the study (Behrens-Fisher *t*-test *P*  = . 005). The drop in anxiety also is evident from the two-way ANOVA, which showed significantly higher anxiety in week one (3.06) than in weeks three or four (1.92 and 1.37, respectively, *P* = . 001). The time series of her anxiety ratings, with the weeks demarcated, are shown in Figure 13. Octavia’s sadness and unhappiness also declined through the course of the study; sadness dropped from a mean of 0.49 to 0.08 (Behrens-Fisher *t*-test *P*  = . 001), and unhappiness dropped from 4.39 to 3.41 (*P* < .001). Her energy ratings on the Mood Map (y-axis) do not reflect the increased energy that she reported, however. As mentioned above, Octavia described improved focus and prioritization over the course of the study. While her anxiety and some other negative moods were lower in the second half of the study, no change in the diurnal pattern of anxiety across the study was revealed by the two-way ANOVA used to simultaneously study diurnal and across-study changes. That is, her anxiety was lower overall by the end of the study, but remained higher in the morning.

#### Eliza

A more complex trend in moods was exhibited by Eliza, a woman in her mid-forties who juggled a full-time job and close relationships with her two sons, husband and extended family. She managed a tight schedule, running each morning, arriving at work by 7:00 AM, picking up her children after school, and reviewing their homework–all before preparing dinner. Historically, Eliza dealt with anxiety and other negative emotions through constant busyness, but said that this coping style eventually left her exhausted. She worked hard to be positive and supportive at work and at home, and experienced deep regret when she let others down. She also described frequent waves of anxiety that “wipe out the joy” of positive moments with her family.

As the study progressed, Eliza expressed great interest in mapping her moods, describing better self-understanding, clearer communication and improved resolution of conflicts with her husband and eldest son. In light of these reported gains, initially it was surprising to see an increased negative affect in her experience sampling data across the study. Specifically, the mean of her energy ratings on the Mood Map decreased from 2.82 to 1.88 from the first to the second half of the study (Behrens-Fisher *t*-test *P* = .007) and her mean sadness ratings increased from 0.69 to 1.38 (*P* = .039). This change, although surprising in light of her reported increased insight and improved communication, made sense on closer analysis. The trend towards negativity in her experience sampling mirrored statements she made in interviews about learning to acknowledge different emotional states. She described calibrating herself on the Mood Map:

I allowed myself more freedom. It’s exploratory. I allowed myself more freedom and range of motion in there just to get myself rolling…I thought, “I’m going to explore what it feels like to put it right over here because that’s where I think I’m at” …[Before] I need[ed] everybody to be happy. This has allowed me to go, ”Oh, it’s okay, I’m not always happy either,”…something I’ve learned from this is, instead of always needing to be in that positive, happy quadrant, accepting that I can be in a negative quadrant, either with energy or with mood, and still be managing myself…that I can be okay even when I’m not in a positive energetic state, and that allows me to say for other people, oh, they can feel that way and still be—I don’t have to fix it.

For Eliza and others, it was difficult to disentangle mood changes from changes in self-awareness. That is, the experience sampling data could reflect either increased distress or acknowledgment of previously disavowed negative moods.

**Figure 14:**
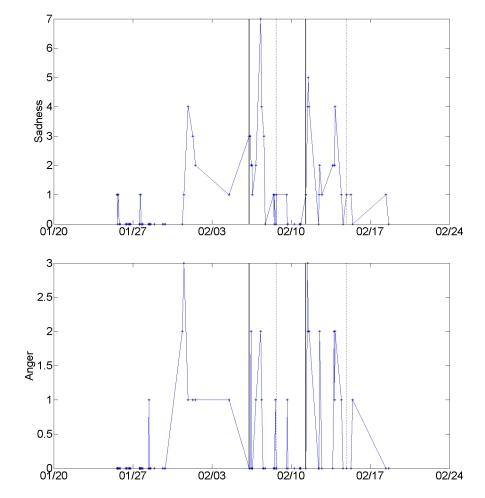
The time series of mood ratings echoes Eliza’s interview account of two stressful episodes; both were family conflicts that stretched over multiple days. The first episode (between 2/6 and 2/8) was characterized in mobile entries as sadness; the second (between 2/11 and 2/14) as sadness and anger. A solid vertical line marks the beginning of each episode, and the end of each episode is marked with a dotted vertical line.

Two stressful episodes, both family conflicts, stood out in Eliza’s interview narratives and experience sampling data (see Figure 14). The first incident occurred shortly before her birthday. Her mother, after trying unsuccessfully to arrange a birthday dinner for Eliza, sent a card, followed by a phone call and an email, all expressing sorrow that they were not able to see one another. Eliza felt a surge of anger after each message, resenting that her mother had manipulated her into feeling guilt. She explained that the mobile therapies helped her sympathize with her mother’s intent and decide to postpone a heated conversation. She also became more comfortable with her decision to decline the dinner invitation and reserve time for herself. Nonetheless, the event took its toll. Eliza described sadness that is mirrored in her experience sampling data: During this episode, her mean ratings for sadness, but no other emotion, rose above the background level (from 0.040 to 1.88, *P* < .001, one-way ANOVA).

The second conflict involved Eliza’s ten-year-old son. A call from his teacher about his disruption of a class triggered her anger: “I was definitely in a rage. I was really angry. I was, you know, I was already at my wit’s end, and I’d been trying to make the afternoon nice and then, you know, all the chemical elements came together.” Shortly after, she regretted lashing out at her son for simply having fun with his friend during class. That afternoon they sat down together, exploring the mobile therapies and scales to process the conflict. Her son started to understand not only his mother’s anger, but also his anger at the teacher. During this episode too, Eliza’s sadness rose significantly above the background level (0.40) to 1.87 (*P*  < .001 one-way ANOVA). In addition, her anger, typically near zero, increased to 0.94 (*P* = .008), and her happiness fell from 5.53 to 4.47 (*P = .*002).

Eliza’s phone entries characterize the first event as more disappointing and the second as more infuriating. She used the mobile therapies for anger management and conflict resolution heavily during both episodes; in fact, one half of her usages of these therapies throughout the study fell on the dates of those episodes. For Eliza, negative affect may have been experienced primarily as sadness, but at a certain threshold developed to include anger.

## Discussion

Emotional awareness and self-regulation are important personal health skills that may be facilitated by mobile technologies. In this exploratory project, we developed and tested a mobile application that enabled users to report their emotional states and access therapeutic exercises based on cognitive behavioral therapy. We examined how people used this application to increase self-awareness in moments of stress, develop insights about their emotional patterns, and practice new strategies for modulating stress reactions.

Interview narratives suggest that study participants applied the mood scales and therapeutic content in ways that helped them initiate meaningful personal change. Examples discussed in the Results section—of a woman who started problem solving in a more collaborative way with her niece and co-worker, a man who became more goal-focused in bureaucratic negotiations,and another who experimented with a new approach to dealing with family stress at the end of his workday—were similar to those shared by other participants. In general, people quickly internalized the mood questions and mobile therapies, applying the concepts whether or not they were physically interacting with the application. Several participants used the concepts from the application to understand and coach other people.

The case studies analyzed self-reflection in weekly interviews in conjunction with experience sampling data. Interview reports of marked patterns (eg, in stress and fatigue) were generally reflected in experience sampling data, with the most striking agreement between the two forms of data appearing in stressful episodes. In these distinct events, participants made clear notations of negative emotions and were far more likely to use mobile therapies than they were at other times. These observations suggest a readiness to use mobile therapies when experiencing intense emotions.

The primary disparity between interviews and experience sampling data was that participants seemed to express more emotional volatility and negativity in the former than in the latter. There are several possible explanations for this disparity. As Kahneman explains in reference to the “peak-end rule,” recall is biased towards intense and recent events [11]. Interviews may have evoked the most dramatic emotional experiences that participants had during the week. Alternatively, people may be more emotionally expressive when interacting with an interviewer than with an application. Some of the disparity appeared to stem from adjusting to the tool over time; for example, one participant who was excited about increased self-awareness reported that she became more comfortable acknowledging negative moods as the study progressed. By highlighting the most emotionally salient events, interviews help in the interpretation of experience sampling data, which includes many data points, equally weighted, over a stretch of time.

This was a preliminary study with limitations that should be addressed in future studies. The first limitation concerns the small sample size used for this initial, qualitative exploration of how people adopt mobile therapies. Our interview approach—repeated, open-ended discussions over the one-month period that participants used the prototype—was influenced by clinical psychology, ethnography, and participatory design. To evaluate the efficacy of such a system, a large controlled study would be required. Second, as noted earlier, a data capture error limited the analysis of the x-axis of the Mood Map to a bipolar distinction between positive and negative responses. Another limitation was the requirement that participants carry an additional phone; future studies should take advantage of application stores, such as those for the iPhone and Android, to test tools on people’s current phones. Ideally the study should continue for a longer period of time to allow accommodation to the mood scales. Finally, evaluating this type of intervention is complicated because it combines therapy and assessment and because people’s use of the tool changes over time.

This study pointed out some key directions for future mobile therapies. The benefits in self-awareness and coping that individuals garnered in this study most likely resulted from a combination of the features on the phone and the reflection offered in weekly interviews. Future systems could combine the assessment, mobile therapies, and feedback that participants experienced in this study. That is, the feedback from interviews could be built into the software and used to customize the mobile therapies. To cultivate self-awareness over daily, weekly, and monthly patterns, the system should ideally present mood trends on the phone immediately after a mood entry. As mentioned above, the system should also invite users to investigate their mood correlates and set goals, activities that were appealing to most of our participants. To help with managing situation-specific stressors, feedback displays could illuminate the contextual triggers and help the user to develop coping strategies. The system could track which therapies were most helpful and provide similar but increasingly sophisticated strategies over time.

In summary, this preliminary study pointed to potential promises of coupling experience sampling tools with mobile therapies to encourage self-awareness and coping in daily life. Future applications should ideally include adaptive learning and query based on an individual’s emotional signature—the range, patterns, and triggers of emotions he or she experiences—and display trends immediately following queries. Mobile therapies should be pushed to the user based on his or her emotional signature, and ideally be integrated with the personal technologies he or she uses for entertainment, calendaring, and communication. We recommend experimental studies to assess the potential benefits of such systems, in addition to larger field deployments to understand how such systems might be adopted in communities. Among other topics that can be examined in qualitative field studies is mood sharing, that is, how people use their Mood Map ratings or media to represent their emotional states, the clusters of people with whom they share mood data, and the contagion effects of mood in social networks.
